# Prevalence and associated factors of suicidal ideation among school-going adolescents in Guyana: results from a cross sectional study

**DOI:** 10.1186/1745-0179-3-13

**Published:** 2007-08-23

**Authors:** Emmanuel Rudatsikira, Adamson S Muula, Seter Siziya

**Affiliations:** 1 Departments of Epidemiology and Biostatistics and Global Health, Loma Linda University, School of Public Health, Loma Linda, California, USA; 2 Department of Community Health, University of Malawi, College of Medicine, Blantyre, Malawi, Africa; 3 Department of Community Medicine, University of Zambia, School of Medicine, Lusaka, Zambia, Africa

## Abstract

**Background:**

Adolescent suicidal behaviour is a neglected public health issue especially in middle- and low-income countries. Informed policy decision-making on suicidal behaviour will need reliable information on the prevalence and correlates of suicidal ideation which is a determinant of suicidal behaviour.

**Methods:**

We estimated the prevalence and associated factors of suicidal ideation among school-going adolescents using data from the Global School-Based Health Survey conducted in 2004 in Guyana.

**Results:**

Of the 1197 respondents, 18.4% (14.9% males and 21.6% females)reported having seriously considered committing suicide in the last12 months. Males were less likely to seriously consider committingsuicide than females (OR = 0.45; 95% CI [0.30, 0.67]). Subjects whoreported having been bullied were more than twice as likely tocontemplate committing suicide as those who had not been bullied (OR = 2.46 [1.71, 3.54]). History of depression was positivelyassociated with suicidal ideation (OR = 2.67; 95% [1.87, 3.81] whilehaving close friends and understanding parents were negativelyassociated with suicide ideation (OR = 0.51; 95% CI [0.28, 0.94] and OR = 0.51; 95% CI [0.35, 0.76] respectively).

**Conclusion:**

Suicidal ideation is a significant public health issue among in-school adolescents in Guyana that requires attention. The design, implementation and evaluation of suicidal behaviour interventions should incorporate our knowledge of these associated factors.

## Background

Suicidal behaviour is an important public health problem worldwide. The World Health Organization (WHO) reports that in 2000, more than 800,000 people died of suicide around the world [1]. Flisher et al. (1993) report that 85.7% of those who make a suicide attempt have seriously thought about doing so earlier [2]. Risk factors for suicidal ideation include depression [3], cigarette smoking [4], alcohol and drug use [5-7], low parental care [8], and experience of bullying [9].

Guyana is a South American country bordering the North Atlantic Ocean, between Suriname and Venezuela. It has an estimated population of 769,000. There are limited data about suicide in Guyana. Much of the available health research literature on this country is on tropical infectious diseases. Hence, the aim of this study is to assess the prevalence and associated factors of suicidal ideation among adolescent students in Guyana.

## Methods

Our study involved secondary analysis of existing data available from the Guyana Global School-Based Health Survey (GSHS) conducted in 2004. The GSHS was developed by the World Health Organization (WHO) in collaboration with United Nations' UNICEF, UNESCO, and UNAIDS with technical assistance from Centers for Disease Control and Prevention (CDC) in Atlanta, USA. The survey aims to provide data on health and other social behaviours among school-going adolescent students.

The GSHS uses a two-stage probability sampling technique. In the first stage, primary sampling units are schools which are selected with a probability proportional to their enrolment size. In the second step classrooms are chosen randomly within a selected school. All the students within the selected classes are eligible for participation. Before administering the survey to teens, parental permission and approval from the Ministry of Health were obtained. Students' participation was anonymous and voluntary. A questionnaire is self-completed anonymously by the students and this takes between 30 to 40 minutes. The school response rate was 100 percent while the student response rate was 80 percent, thus making overall response rate 80 percent.

Our main outcome variable was self-reported history of suicidal ideation within the past 12 months. Study participants were asked the question: During the past 12 months, did you ever seriously consider attempting suicide? Data were also collected on age, gender, current cigarette smoking, having been bullied, alcohol use, depression, self assessment of parental supervision and having friends. To assess depression, we considered responses to the following question: "During the past 12 months, did you ever feel so sad or hopeless almost every day for two weeks or more in a row that you stopped doing some usual activities?"

A weighting factor was used in the analysis to reflect the likelihood of sampling each student and to reduce bias by compensating for differing patterns of non response. The weight used for estimation is given by the following formula:

W = W1 * W2 * f1 * f2 * f3 * f4,

where W1 = the inverse of the probability of selecting the school

W2 = the inverse of the probability of selecting the classroom within the school

fl = a school-level non response adjustment factor calculated by school size category (small, medium, large)

f2 = a class-level non response adjustment factor calculated for each school

f3 = a student-level non response adjustment factor calculated by class

f4 = a post stratification adjustment factor calculated by grade.

We conducted logistic regression analysis using SUDAAN software version 9.0 (Research Triangle Institute, NC, USA) to estimate the association between relevant predictor variables and suicidal ideation in the last 12 months. We report unadjusted Odds Ratios for selected predictor variables while considering suicidal ideation in the last 12 months as a dependent variable. We thereafter report results of adjusted odds ratios for the factors, having controlled for factors identified as significant in the bivariate analysis. An α of 0.05 was used in both bivariate and multivariate analyses.

## Results

Table 1 presents selected characteristics of the study population of 1197 Guyanese adolescents. Most of the sample was female (51.0%), 14 years old (30.3%), non smokers, non alcohol drinkers and with understanding parents (78.1%). Overall 18.4% (14.9% males and 21.6% females) had seriously considered suicide in the last 12 months.

**Table 1:** Selected socio-demographic characteristics of the study population in the Guyana Global School-Based Health Survey, 2004

	Total 100 (1197)	Males 49.0 (504)	Females 51.0 (693)
Age (years)			
<14	25.8 (302)	24.1 (115)	27.4 (187)
14	30.3 (378)	32.4 (165)	28.3 (213)
15	32.5 (386)	28.7 (146)	36.2 (240)
≥16	11.4 (131)	14.8 (78)	8.1 (53)
Smoking			
No	92.9 (1077)	89.2 (429)	96.4 (648)
Yes	7.1 (75)	10.8 (51)	3.6 (24)
Alcohol use			
No	55.3 (684)	43.6 (226)	66.5 (458)
Yes	44.7 (513)	56.4 (278)	33.5 (235)
Bullied			
No	61.1 (664)	59.2 (269)	62.9 (395)
Yes	38.9 (410)	40.9 (182)	37.1 (228)
Depression			
No	67.4 (789)	71.1 (347)	64.1 (442)
Yes	32.6 (385)	29.0 (140)	35.9 (245)
Suicidal ideation			
No	81.6 (953)	85.1 (417)	78.4 (536)
Yes	18.4 (219)	14.9 (72)	21.6 (147)
Close friends			
No	9.2 (106)	8.6 (42)	9.8 (64)
Yes	90.8 (1091)	91.4 (462)	90.2 (629)
Understanding parents			
No	22.0 (258)	21.7 (108)	22.2 (150)
Yes	78.1 (912)	78.3 (381)	77.8 (531)

Table 2 indicates that male subjects were less likely to contemplate suicide than females (OR = 0.64; 95% CI [0.46, 0.87]). For both males and females, suicide ideation was associated with drinking alcohol (OR = 2.09; 95% CI [1.21, 3.61] for males and OR = 1.93; 95% CI [1.31, 2.83] for females), having been bullied (OR = 2.25; 95% CI [1.26, 3.61] for males and OR = 3.62; 95% CI [2.42, 5.42] for females) and depression (OR = 2.25; 95% CI [1.26, 40.2] for males and OR = 3.62; 95% CI [2.42, 5.42] for females). Smoking was positively associated with suicidal ideation in females (OR = 3.25; 95% CI [1.41, 7.50]). Having close friends and understanding parents were negatively associated with suicide ideation (OR = 0.58; 95% CI [0.35, 0.95] and OR = 0.45; 95% CI [0.32, 0.62] respectively).

**Table 2:** Factors associated with suicidal ideation among adolescents in Guyana, 2004

	Unadjusted odds ratios with 95% CI		
	Total	Males	Females

Age (years)			
<14	1.00	1.00	1.00
14	0.88 [0.59, 1.31]	0.82 [0.42, 1.60]	0.91 [0.56, 0.49]
15	0.85 [0.58, 1.26]	0.73 [0.37, 1.43]	0.90 [0.56, 1.46]
≥16	0.70 [0.39, 1.26]	0.41 [0.16, 1.03]	1.28 [0.59, 2.75]
Gender			
Females	1.00	-	-
Males	0.64 [0.46, 0.87]	-	-
Close friends			
No	1.00	1.00	1.00
Yes	0.58 [0.35, 0.95]	0.43 [0.20, 0.96]	0.72 [0.38, 1.34]
Smoking			
No	1.00	1.00	1.00
Yes	1.85 [1.08, 3.19]	1.66 [0.77, 3.56]	3.25 [1.41, 7.50]
Alcohol use			
No	1.00	1.00	1.00
Yes	1.70 [1.25, 2.30]	2.09 [1.21, 3.61]	1.93 [1.31, 2.83]
Bullied			
No	1.00	1.00	1.00
Yes	2.87 [2.06, 3.99]	2.25 [1.26, 4.02]	3.62 [2.42, 5.42]
Depression			
No	1.00	1.00	1.00
Yes	2.87 [2.06, 3.99]	2.25 [1.26, 4.02]	3.62 [2.42, 5.42]
Understanding parents			
No	1.00	1.00	1.00
Yes	0.45 [0.32, 0.62]	0.50 [0.29, 0.88]	0.41 [0.27, 0.62]

Table 3 presents results from multivariate analysis. Male gender, having close friends and understanding parents remained negatively associated with suicidal ideation. Likewise, having been bullied and depression remained positively associated with suicidal ideation.

**Table 3:** Factors associated with suicidal ideation among adolescents in Guyana, 2004

Variable	Adjusted odds ratios with 95% CI
Age (years)	
<14	1.00
14	0.98 [0.60, 1.58]
15	0.76 [0.49, 1.24]
≥16	0.96 [0.50, 1.83]
Gender	
Females	1.00
Males	0.45 [0.30, 0.67]
Close friends	
No	1.00
Yes	0.51 [0.28, 0.94]
Smoking	
No	1.00
Yes	1.64 [0.82, 3.27]
Alcohol	
No	1.00
Yes	1.37 [0.68, 2.77]
Bullied	
No	1.00
Yes	2.46 [1.71, 3.54]
Depression	
No	1.00
Yes	2.67 [1.87, 3.81]
Understanding parents	
No	1.00
Yes	0.51 [0.35, 0.76]

## Discussion

Our study found that overall 18.4% (14.9% males and 21.6% females) had seriously considered suicide in the last 12 months. Our estimates are much higher than the prevalence of suicidal ideation among Americans of Caribbean descent (10.7%) [10], but similar to rates of suicidal ideation in Brazil (17.1%) [11]. Previous studies have reported similar results with regard to female adolescents having a higher prevalence of suicidal ideation than males [12-14].

The high rate of suicidal ideation in Guyana can be explained in part by poverty and high prevalence of HIV/AIDS in the country. Guyana is one of the poorest countries in the Caribbean region with $1,029 GDP per capita [15] and ranks third in the prevalence of HIV/AIDS [16]. In a study of risk and resilience factors of suicidality among Latino and African American youth, O'Donnell et al found that those with unmet needs were at greater risk of suicidal ideation [17]. Several studies have reported the association between suicidal behaviours and HIV/AIDS [18-20]. This is primarily due to social isolation and hopelessness.

Results from this study indicate that history of current smoking, alcohol use, depression, having been bullied and being females were associated with higher odds of having experienced suicidal ideation. Several studies indicate that suicidal ideation is positively associated with depression [2,15,21], alcohol and substance abuse [4,5,7]. In a study of 2341 US high school students, Klomek et al found that victims of bullying were more than five times likely to have seriously considered committing suicide than those who were not bullied.

In this study, adolescents who had close friends and understanding parents were less likely to report having thought of committing suicide in the past 12 months. Chiou et al report that parent-child conflict is one of the most common precipitating factors of suicide attempts [21]. Heider et al have reported that among adults in the European Study of Epidemiology of Mental Disorders project, suicidality was associated with low parental care as a child [22]. Fortuna et al report that among Latino subgroups in United tates, family conflict is independently associated with suicide attempts [23]. Cheng and Chan indicate that support from family and friends lower suicidality by reducing death acceptability and stress [24].

Our study had a number of limitations. Data collection used a self-completed questionnaire. There is therefore possibility that some study participants may have misreported on our outcome of interest or the predictor variables. The study also only recruited adolescents in school. To the extent that in-school adolescents differ from out of school adolescents, our finding may not be applicable to all adolescents in Guyana. Also since data used in this study are from a cross sectional survey, we can not ascribe causation to any of the factors that were identified as associated with suicidal ideation.

## Conclusion

We found a 12-month overall prevalence of suicidal ideation among school-going adolescents in Guyana at 18.4%. Factors associated with the outcome were female gender, having being bullied, alcohol use, cigarette smoking and depression. Having a supporting environment with friends and understanding parents was associated with lower odds of having contemplating suicide. Public health intervention aimed to reduce adolescent suicidal behaviours should factor in the socio-demographic correlates of suicidal ideation. The clustering of multiple unhealthy lifestyles and co-morbid conditions such as cigarette smoking, alcohol use and depression is of public health concern.

## Abbreviations

CDC- Centers for Disease Control and Prevention

GSHS- Global School-Based Health Survey

HIV- Human immunodeficiency virus

OR- Odds ratio

WHO- World Health Organisation

UNAIDS- Joint United Nations' HIV/AIDS Programme

UNESCO- United Nations Educational, Scientific and Cultural Organisation

UNICEF- United Nations Children' Fund

## Competing interests

The author(s) declare that they have no competing interests.

## Authors' contributions

ER conducted data analysis, participated in interpretation of finding and writing of manuscript.

ASM participated in interpretation of findings and drafting manuscript.

SS participated in interpretation of findings and drafting of manuscript.

All authors read and approved the final draft of the manuscript.
